# Hypoxia Stimulates SUMOylation-Dependent Stabilization of KDM5B

**DOI:** 10.3389/fcell.2021.741736

**Published:** 2021-12-17

**Authors:** Bingluo Zhou, Yiran Zhu, Wenxia Xu, Qiyin Zhou, Linghui Tan, Liyuan Zhu, Hui Chen, Lifeng Feng, Tianlun Hou, Xian Wang, Dingwei Chen, Hongchuan Jin

**Affiliations:** ^1^ Laboratory of Cancer Biology, Key Lab of Biotherapy in Zhejiang Province, Cancer Center of Zhejiang University, Sir Run Run Shaw Hospital, School of Medicine, Zhejiang University, Hangzhou, China; ^2^ Department of Medical Oncology, Sir Run Run Shaw Hospital, School of Medicine, Zhejiang University, Hangzhou, China; ^3^ Department of Pathology, Sir Run Run Shaw Hospital, School of Medicine, Zhejiang University, Hangzhou, China; ^4^ Department of Clinical Medicine, Wenzhou Medical University, Wenzhou, China; ^5^ Department of General Surgery, Sir Run Run Shaw Hospital, School of Medicine, Zhejiang University, Hangzhou, China

**Keywords:** hypoxia adaption, GC, KDM5B, SUMOylation, PIAS4, ubiquitination

## Abstract

Hypoxia is an important characteristic of the tumor microenvironment. Tumor cells can survive and propagate under the hypoxia stress by activating a series of adaption response. Herein, we found that lysine-specific demethylase 5B (KDM5B) was upregulated in gastric cancer (GC) under hypoxia conditions. The genetic knockdown or chemical inhibition of KDM5B impaired the growth of GC cell adapted to hypoxia. Interestingly, the upregulation of KDM5B in hypoxia response was associated with the SUMOylation of KDM5B. SUMOylation stabilized KDM5B protein by reducing the competitive modification of ubiquitination. Furthermore, the protein inhibitor of activated STAT 4 (PIAS4) was determined as the SUMO E3 ligase, showing increased interaction with KDM5B under hypoxia conditions. The inhibition of KDM5B caused significant downregulation of hypoxia-inducible factor-1α (HIF-1α) protein and target genes under hypoxia. As a result, co-targeting KDM5B significantly improved the antitumor efficacy of antiangiogenic therapy *in vivo*. Taken together, PIAS4-mediated SUMOylation stabilized KDM5B protein by disturbing ubiquitination-dependent proteasomal degradation to overcome hypoxia stress. Targeting SUMOylation-dependent KDM5B upregulation might be considered when the antiangiogenic therapy was applied in cancer treatment.

## Introduction

Cancer ranks as a leading cause of death and an important barrier to extend life expectancy in every country of the world. According to the report recently released by the International Agency for Research on Cancer, GC remains one of the most common causes of cancer death worldwide. Although multidisciplinary treatment has made big progress (Joshi and Badgwell, 2021), GC still ranked fifth for incidence and fourth for mortality globally (Sung et al., 2021). The understanding of the pathogenesis and development mechanism of GC is attracting considerable attention in order to find effective therapeutics and prevention strategies.

Hypoxia is a state of low oxygen tension which is common in numerous solid tumors typically associated with abnormal vasculature (Sharma et al., 2019). As a result, tumor cells have to undergo adaptive genetic or epigenetic changes resulting into metabolic remodeling, angiogenesis, or invasiveness to overcome multiple hypoxia-associated challenges (Patel and Sant, 2016). By doing so, tumor cells adapted to hypoxia have a growth advantage and become resistant to chemotherapy or radiotherapy, conferring worse prognosis (Hammond and Giaccia, 2006). More importantly, antiangiogenesis has been widely explored and applied in the clinical management of various cancers as a new targeted therapy (Itokawa et al., 1991; Ohtsu et al., 2011; Fuchs et al., 2014). Co-targeting hypoxia adaption would be essential to improve such antiangiogenesis therapies.

Histone lysine demethylases, responsible for histone demethylation, have attracted extensive research interests recently (Kooistra and Helin, 2012; Højfeldt et al., 2013). Among them, KDM5B has been reported to be relevant in multiple human cancers including GC (Xhabija and Kidder, 2019). We have previously reported that KDM5B promoted GC chemoresistance by demethylating H3K4 to facilitate the recruitment of XRCC1 for efficient repair of DNA damage (Xu et al., 2018). And it was also confirmed to be overexpressed in GC and required for proliferation and metastasis *in vivo* and *in vitro* (Wang et al., 2015; Bao et al., 2016; Li et al., 2019).

Post-translational modification (PTM) of proteins is a rapid and economical manner to regulate protein expression and function in response to various stimuli. However, PTM and functions of KDM5B have not been well defined. It was reported that the phosphorylation (Yeh et al., 2019) or SUMOylation (Bueno and Richard, 2013) of KDM5B attenuated its occupancy on the promoters of target genes. KDM5B can also be ubiquitinated by E3 ligase RNF4 for subsequent proteasomal degradation during the cell cycle (Bueno and Richard, 2013) or in response to DNA damages (Hendriks et al., 2015). However, the regulation and relevance of PTM of KDM5B under hypoxia remain largely undefined.

In this study, we found another SUMO E3 ligase PIAS4 mediated KDM5B SUMOylation under hypoxia to protect it from ubiquitination-dependent proteasomal degradation, which was important for the hypoxia adaption of GC cells. Genetic or chemical inhibition of KDM5B can disrupt hypoxia adaption both *in vitro* and *in vivo*. Thus, targeting KDM5B represents a new combination option for antiangiogenesis or other hypoxia-inducing therapeutics.

## Materials and Methods

### Cell Culture and Small-Molecule Chemical Inhibitors

Nine human GC cell lines (SGC7901, BGC823, MKN45, AGS, HGC-27, MKN28, MGC803, N87, and MFC), a normal human gastric epithelial cell line (GES-1), and HEK293T were all purchased from the Cell Bank of the Typical Culture Preservation Committee, Chinese Academy of Sciences (Shanghai, China). MEF cells from *Pias4* ^*+/+*^ and *Pias4* ^*−/−*^ mice were generous gifts from Prof. Shuai Ke (University of California, Los Angeles, CA, United States) (Yu et al., 2018). SGC7901/BGC823-shNC and SGC7901/BGC823-shKDM5B cells were generated via the infection of lentiviral vectors containing shRNA (shscramble and shKDM5B) and puromycin selection. The cells were cultured according to the culture conditions previously reported (Xu et al., 2019).

The chemicals used in this study include JIB04 (S7281, Selleck), 2-D08 (S8696, Selleck), TAK-981 (S8829, Selleck), ADOX (S8608, Selleck), MS049 (S8147, Selleck), AMI-1 (S7884, Selleck), EX527 (S1541, Selleck), TSA (S1045, Selleck), A485 (S8740, Selleck), C646 (S7152, Selleck), okadaic acid (OA) (S1786, Beyotime), MG132 (474790, Calbiochem, United States), CHX (C7698, Sigma-Aldrich), and NEM (Sigma-Aldrich, E3876), CoCl_2_ (Sigma-Aldrich, C8661). The antibodies used were listed as follows: anti-KDM5B (Abcam ab181089, for WB), anti-KDM5B (Abcam ab211366, for IHC), anti–β-actin (Abclonal ac026), anti-PIAS4 (CST 4392s), anti-FLAG-tag (Sigma-Aldrich F1804-1), anti–HA-tag (earthox, E022010), anti–V5-tag (Invitrogen 46-0705), anti–p-RB (CST 8516S), anti-Rb (santa cruz sc-102), anti-p21 (CST 2946S), anti-cyclin E (CST 20808), anti-UB (Santa cruz sc-8071), anti-His (Proteintech 66,005-1-ig), anti-CD31 (Abcam ab24590), anti-CA9 (Proteintech 11071-1-AP), anti-HIF1α (CST 36169S, for WB), and anti-HIF1α (NB100-105, for IHC).

### SiRNA and Plasmids

Small interfering RNA (siRNA)–targeting KDM5B, PIAS1, PIAS2, PIAS3, and PIAS4 were synthesized by Genepharma company (Shanghai, China). The sequences of these siRNAs are listed in Supplementary Table S1. The siRNAs were transfected into the cells seeded overnight using the Lipofectamine RNAiMAX transfection reagent (Invitrogen, United States) according to the manufacturers’ instruction.

The plasmid of FLAG-KDM5B was constructed by GeneChem Company (Shanghai, China). The plasmid of PIAS4-HA tag was purchased from Sino Biologic Inc. (Beijing, China). SUMO1/2/3 ORFs with V5 or His tag were cloned to the pCMV3 vector. The QuikChange Site-Directed Mutagenesis Kit (Agilent, United States) was used to generate PIAS4 mutants and non-conjugation SUMO mutants SUMO1/2/3ΔGG. The primers used are shown in Supplementary Table S2. Plasmids were transfected into the cells that were seeded overnight with the X-tremeGENE HP DNA Transfection Reagent (Roche, Basel, Switzer-land) according to the instruction provided. After transfection for 48–72 h, the cells were harvested for RNA or protein extraction and other assays.

### RNA Isolation and Quantitative Real-Time PCR

Total RNA was extracted by the Trizol reagent (Invitrogen, United States). The concentration of total RNA was quantified by NanoDrop 2000. And 2 μg of total RNA was taken for reverse transcription using the High-Capacity cDNA Reverse Transcription Kit (Applied Biosystems, United States). The relative expression level of each mRNA was determined by qPCR with the SYBR Green Master reagents (Applied Biosystems, United States). The primers were displayed in Supplementary Table S3.

### Western Blotting

Proteins from the cells were extracted by RIPA lysis buffer (Beyotime, Beijing, China) and quantified by the BCA Protein Assay Kit (Beyotime, Beijing, China). Lysates with 20–30 μg protein were loaded to SDS–PAGE gel for separating proteins of different molecular weights and transferred to the PVDF membranes. The membranes were blocked with 5% skimmed milk dissolved in TBST for 1 h. Subsequently, the membranes were incubated with the primary antibody overnight at 4°C, followed by incubation with the secondary antibody labeled with peroxidase (Jackson ImmunoResearch Inc., PA, United States) for 1 h at room temperature. The WB bands were visualized with the ECL substrate.

### Cell Viability Assay

Cells were seeded in a 96-well plate and treated as indicated. After incubation with CCK8 dye, cell viability was measured with a BioTek Gen5 microplate spectral photometer at OD 450 nm.

### Immunoprecipitation

Cells were washed with ice-cold PBS twice and lysed on ice in 20 mM Triton buffer containing 50 mM Tris–HCl (PH 7.4), 150 mM NaCl, 1% Triton X-100, and protease inhibitor cocktail. After taking the input, the lysates were incubated with primary antibodies overnight at 4°C. On the next day, protein A/G sepharose beads were added into the lysates. After rotation at 4°C for 3 h, the beads were washed with lysis buffer 5 times. The bound proteins were then eluted and detected by Western blotting, as described previously.

### Chromatin Immunoprecipitation

ChIP analysis was implemented with the SimpleChIP™ Enzymatic Chromatin IP Kit (CST, United States) according to the protocol provided, using the antibody anti–tri-methylation H3K4 (CST, 1: 100) and negative control anti-IgG. The primers used for the PCR analysis of the precipitated DNA are shown in Supplementary Table S3.

### Immunohistochemistry

The tumors from mice with the indicated treatment and tissues from GC patients in the tissue microarray were fixed with 4% paraformaldehyde. For IHC staining, the tissues were incubated with diluted primary antibodies at 4°C overnight and with biotinylated secondary antibody, followed by 3,3′-diaminobenzidine (DAB) and hematoxylin staining on the next day. The results were observed and analyzed by a professional pathologist. Digital images of five random regions were taken for each tissue section. The H-score was applied to quantify the analysis result, which was calculated using the following formula: H-score = %cells (1, <10%; 2, 10–50%; 3, 50–80%; 4, >80%) * intensity (0, negative; 1, +; 2, 2+; 3, 3+). The intratumoral microvascular density (MVD) was evaluated with CD31 staining. The Chalkley counting method was used to assess the mean number of vessels at ×200 magnification of each section, as described previously (Mohammed et al., 2007).

### Human Gastric Tissue Specimens

The GC and corresponding normal tissues for IHC were all collected from Sir Run Run Shaw hospital (Hangzhou, China) with informed consent obtained from all individuals.

### Ni-Beads Pull-Down Assay

To determine the ubiquitination of KDM5B, HEK293T cells were co-transfected with plasmids including His-HA-UB or His-SUMO1/2/3 for 48 h, as indicated. Subsequently, the cells were treated with MG132 (20 μM) for 6 h and then lysed in the solution, as previously reported (Zhou et al., 2019). Then, KDM5B-UB or KDM5B-SUMO was pulled down by the Ni-beads and washed. The proteins were eluted and analyzed by Western blotting, as described before.

### SUMOylation Assay

To detect the SUMOylation of KDM5B, the protocol (Barysch et al., 2014) was followed as reported before. In brief, HEK293T cells were co-transfected with plasmids as indicted for 48 h. The cells were lysed with 2X lysis buffer (1X PBS, 2% (wt/vol) SDS, 10 mM EDTA, 10 mM EGTA, and 10 mM NEM) at room temperature, followed by sonication twice using a microtip (25 pulses with 30 duty cycles and output control of 3). And 50 μl DTT solution (50 mM final concentration) was added to the samples and boiled at 97°C for 10 min. Then 1/10 lysate was obtained as input before diluting the lysate with 1% Triton buffer with NEM as described in the immunoprecipitation assay (1:10 dilution). After centrifuging at 16,000 g for 15 min, the supernatant was incubated with the FLAG antibody by rotating at 4°C overnight and later with the same steps as described for the immunoprecipitation assay.

### Plate Colony Formation Assay

The cells as indicated were seeded into 6-well plates at a concentration of 500 cells per well. The cells were subsequently incubated with the treatment as indicated for 10 days. The individual colonies with more than 50 cells were counted and qualified with the crystal violet staining.

### Flow Cytometry Analysis

The cell cycle was determined using the cell cycle staining kit (Multi Sciences, Hangzhou, China) using propidium iodide (PI) staining. And the apoptosis was determined by the apoptosis staining kit (Multi Sciences, Hangzhou, China) with PI and Annexin-V FITC.

### *In situ* Proximity Ligation Assay

PLA was performed using the Duolink kit (Sigma) following the manufacturer’s instructions. HEK293T were co-transfected with FLAG-KDM5B and HA-PIAS4, and primary mouse anti-FLAG and primary rabbit anti-PIAS4 were applied to the fixed and permeabilized cells for 4 h at 4°C. After washing, PLUS and MINUS PLA probes provided by the kit were incubated with the primary antibodies for 1 h in the humidity chamber at 37°C. Then, the probes were washed away, and the cells were incubated in ligation and amplification buffer in the kit sequentially in the humidity chamber at 37°C. The slide were then mounted with DAPI and sealed with a coverslip. The PLA signals were visualized as red fluorescent spots.

### Tumor Xenograft Assay

Five-week-old female BALB/c nude mice from the Center of Experimentation of Zhejiang University were fed and housed. And the procedures were performed in compliance with the NIH Guide for the Care and Use of Laboratory Animals. Tumors were established by subcutaneous injection (5 × 10^6^ MFC cells in 0.1 ml saline) into the flanks of the mice. When the tumors exceeded 100–150 mm^3^ in size, the mice were evenly distributed into groups as indicated (n = 6/group) with treatment of JIB04 (55 mg/kg body weight; i.g, every 2 days), 2-D08 (5 mg/kg body weight; i.p, every 2days), Endostar (8 mg/kg body weight; i.p, every 2 days), or equal volume of solvent as indicated at the same time, respectively. The volume of tumors and body weight were measured every 2 days. The mice were euthanized when the tumors size reached approximately 1,500 mm^3^. And the volume of tumors was calculated using the following formula: (length × width^2^)/2.

### Statistical Analysis

All data were presented as mean ± SD, and all experiments were performed at least three times independently. The statistical approach used in every experiment to compare the differences between groups is given in Figure legends. *p* < 0.05 was respected as statistically significant.

## Results

### KDM5B Was Upregulated in GC

To investigate the involvement of KDM5B in GC, we examined KDM5B expression by immunohistochemical staining in tissue microarrays (TMAs) containing collected tumor tissue samples and the matched normal adjacent tissues from 71 GC patients. As shown in Supplementary Figures S1A,B, the expression of KDM5B was much higher in cancer tissues than normal tissues. Next, we determined KDM5B protein expression by Western blotting in cell lines. Consistent with immunohistochemical staining results, KDM5B expression was upregulated in GC cell lines compared to the human normal gastric epithelial cell line GES-1 (Supplementary Figure S1C). Importantly, both plate colony formation and CCK8 assay verified that KDM5B knockdown significantly inhibited the cell proliferation of GC cells (Supplementary Figures S1D–H). Similarly, the chemical inhibitor JIB04, a selective JmjC histone demethylase inhibitor (Bayo et al., 2018), could significantly inhibit the proliferation of GC cells (Supplementary Figures S1I–M). Taken together, these results suggest that KDM5B is upregulated to stimulate cell proliferation in GC.

### KDM5B Inhibition Upregulated p21 Expression

To further explore the underlying mechanism of growth inhibition induced by KDM5B inhibition, we performed RNA sequencing on JIB-04–treated GC cells. The cluster analysis showed that the gene expression profile was significantly changed after JIB-04 treatment (Supplementary Figure S2A). The Kyoto Encyclopedia of Genes and Genomes (KEGG) pathway analysis indicated that differential expressed genes (DEGs) were enriched in “‘cell cycle” and “apoptosis” pathways (Supplementary Figure S2B). Therefore, flow cytometry was used to detect cell cycle distribution with KDM5B knockdown or chemical inhibition. Interestingly, flow cytometry analysis revealed G1 phase arrest after JIB04 treatment (Supplementary Figure S2C) and KDM5B knockdown (Supplementary Figure S2D). Mechanistically, we focused on 18 differentially expressed genes related to cell cycle regulation (Supplementary Figure S2E). After verification by qPCR, p21 turned out to be the most significantly upregulated gene (Supplementary Figure S2F), which was consistent with the phenotype observed in KDM5B stably knockdown cells (Supplementary Figures S2G, H). It is well known that KDM5B could reduce H3K4 tri-methylation to repress gene transcription (Xhabija and Kidder, 2019). As expected, chromatin immunoprecipitation (ChIP) analysis indeed revealed increased tri-methylation H3K4 occupancy at the p21 promoter upon KDM5B inhibition (Supplementary Figures S2I–K). Consistently, p21 protein was significantly upregulated, while the levels of phosphorylated RB, a G1 phase-related regulator was significantly decreased (Supplementary Figure S2L). Once p21 was knocked down, the effect of KDM5B inhibition on G1 arrest was partly reversed (Supplementary Figures S2M,N). Overall, these results indicate that KDM5B promotes H3K4 demethylation to repress p21 transcription, thus resulting in G1 arrest.

### KDM5B Protein Was Upregulated to Facilitate Hypoxia Adaption

**FIGURE 1:**
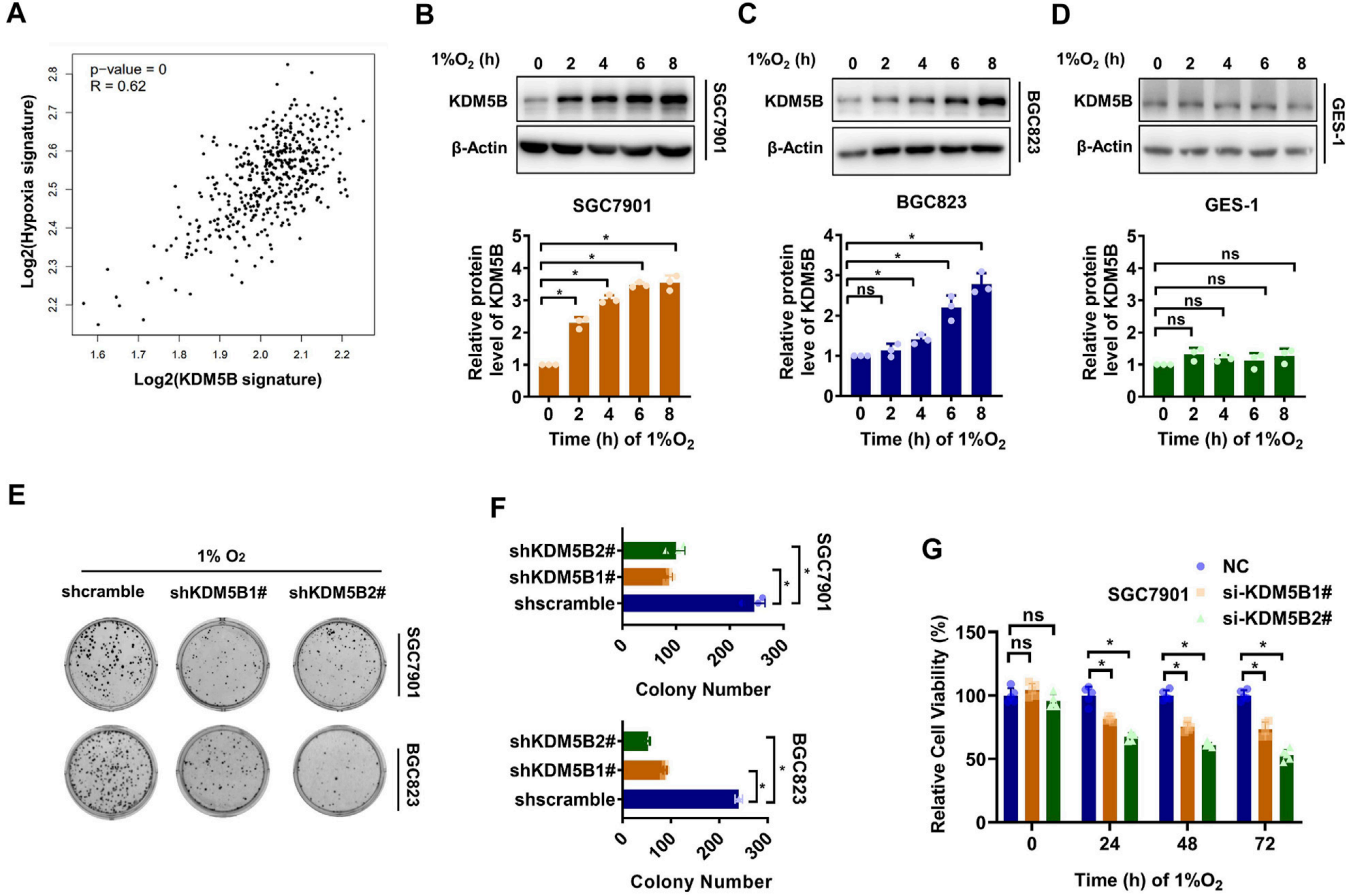
KDM5B protein was upregulated to facilitate hypoxia adaption. **(A)** The correlation of 1,267 putative KDM5B-dependent genes and hypoxia signature was detected by GEPIA. **(B–D)** KDM5B protein level in SGC7901 **(B)**, BGC823 **(C)**, and GES-1 **(D)** cells cultured under hypoxia (1% O_2_) as indicated was determined by Western blotting **(upper panel)**. The relative KDM5B protein level normalized by 0 h was calculated from three individual experiments, after scanning the blot with ImageJ software and quantifying relative to β-Actin **(down panel)**. **(E and D)** Plate colony formation assay for SGC7901 and BGC823 cells before and after KDM5B knockdown were evaluated under hypoxia conditions (1% O_2_) (mean ± SD, n = 3, one-way ANOVA, *p < 0.05). **(G)** CCK8 assay was applied to measure the relative viability of SGC7901 cells under hypoxia conditions (1% O_2_) at 24, 48, and 72 h after KDM5B knockdown (mean ± SD, n = 3, one-way ANOVA, *p < 0.05).

Cancer cells undergo a variety of biological responses to enable cellular survival under various stresses such as hypoxia. Interestingly, the expression of KDM5B signature which was defined by genes significantly down-regulated after JIB04 treatment correlated positively with the expression of the previously reported hypoxia signature (Harris, 2002), based on the TCGA data analysis (Figure 1A). Indeed, KDM5B protein expression was increased significantly as early as 2 h after hypoxia induction in SGC7901 (Figure 1B) and 4 h in BGC823 (Figure 1C) cells but not the non-tumor gastric epithelial GES-1 cells (Figure 1D). Furthermore, both the colony formation (Figures 1E,F) and viability (Figure 1G) of GC cells were impaired significantly under hypoxia conditions once KDM5B expression was knocked down. Taken together, GC cells upregulate the KDM5B protein level to adapt hypoxia.

### Hypoxia Induced SUMO3-Dependent SUMOylation and Subsequent Stabilization of KDM5B

**FIGURE 2:**
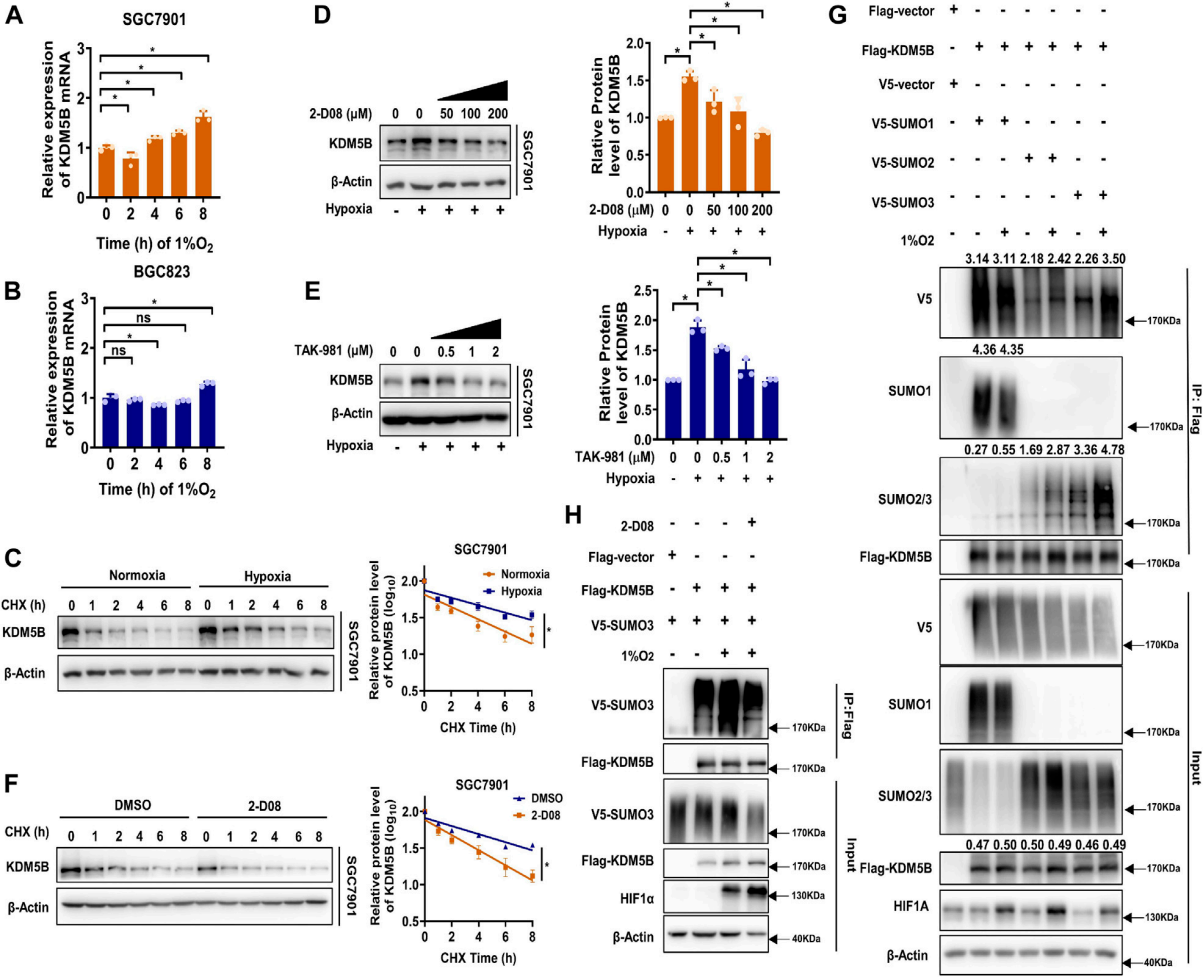
Hypoxia induced SUMO3-dependent SUMOylation and subsequent stabilization of KDM5B. **(A and B)** qPCR analysis of KDM5B mRNA expression in SGC7901 **(A)** and BGC823 **(B)** cells at indicated time points under hypoxia (1% O_2_) (mean ± SD, n = 3, one-way ANOVA, *p < 0.05). **(C)** The half-life of KDM5B protein in SGC7901 cells under normoxia and hypoxia conditions (1% O_2_) was determined by CHX (50 μM) assay. The relative KDM5B protein level was measured by ImageJ, and semi-log plots were shown on the right panel (*mean ± SD, n = 3,* ANCOVA analysis, *p < 0.05). **(D and E)** SGC7901 cells treated with 2-D08 **(D)** or TAK-981 **(E)** as indicated concentration for 18 h before 6 h 1% O_2_ treatment; the KDM5B protein level was determined by Western blotting, the quantification of KDM5B relative to β-Actin was performed and normalized by the normoxia group **(right panel)**. **(F)** The half-life of KDM5B protein in SGC7901 cells under hypoxia (1% O_2_) with 2-D08 (200 μM) or DMSO treatment was determined by the CHX assay. The relative KDM5B protein level was quantified by ImageJ, and the curve was plotted **(right panel)** (*mean ± SD, n = 3,* ANCOVA analysis, *p < 0.05). **(G)** HEK293T cells were co-transfected with indicated plasmids for 48h, and the SUMOylation assay was performed with or without 6 h 1%O_2_ treatment. **(H)** HEK293T cells were co-transfected with FLAG-KDM5B and V5-SUMO3 for 48 h before 2-D08 (200 μM) treatment for another 24 h were subjected to the SUMOylation assay.

Much to our surprise, KDM5B mRNA expression was not altered as strikingly as KDM5B protein (Figures 2A,B) under hypoxia. Meanwhile, the half-life of KDM5B protein was obviously extended under 1% O_2_ treatment (Figure 2C, Supplementary Figure S3A). Therefore, hypoxia seems to stabilize the KDM5B protein. Given that the PTM has been regarded as a key factor affecting protein stability (Buuh et al., 2018), we screened the effect of common PTMs including SUMOylation, acetylation, methylation, and phosphorylation on KDM5B protein stability with corresponding inhibitors (Supplementary Figure S3B). Only 2-D08, the inhibitor of SUMOylation (Kim et al., 2014), could remarkably decrease KDM5B protein level, implicating the involvement of SUMOylation on the regulation of KDM5B protein stability. In fact, the increase in the KDM5B protein level under hypoxia was compromised by 2-D08 dose dependently (Figure 2D). And a similar effect was observed with another SUMOylation inhibitor TAK-981 (Langston et al., 2021) (Figure 2E).Furthermore, SUMOylation inhibition significantly shortened the half-life of KDM5B protein in both SGC7901 and BGC823 cells under hypoxia (Figure 2F, Supplementary Figure S3C). These results indicated that SUMOylation might directly or indirectly affect the stability of KDM5B protein.

Indeed, KDM5B protein could be modified by SUMO1/2/3 but not the non-conjugation of SUMO mutants (SUMO1/2/3ΔGG) (Yuan et al., 2010) (Supplementary Figure S3D). The SUMO2- and SUMO3-mediated SUMOylation were enhanced by hypoxia, but the increase in SUMO3 was more significant (Figure 2G), and 2-D08 dramatically diminished SUMO3-mediated SUMOylation induced by hypoxia (Figure 2H). Collectively, SUMO3-mediated SUMOylation of KDM5B was enhanced under hypoxia stress and increased the stability of KDM5B protein.

### PIAS4 Was the SUMO E3 Ligase for Hypoxia-Induced KDM5B SUMOylation

**FIGURE 3:**
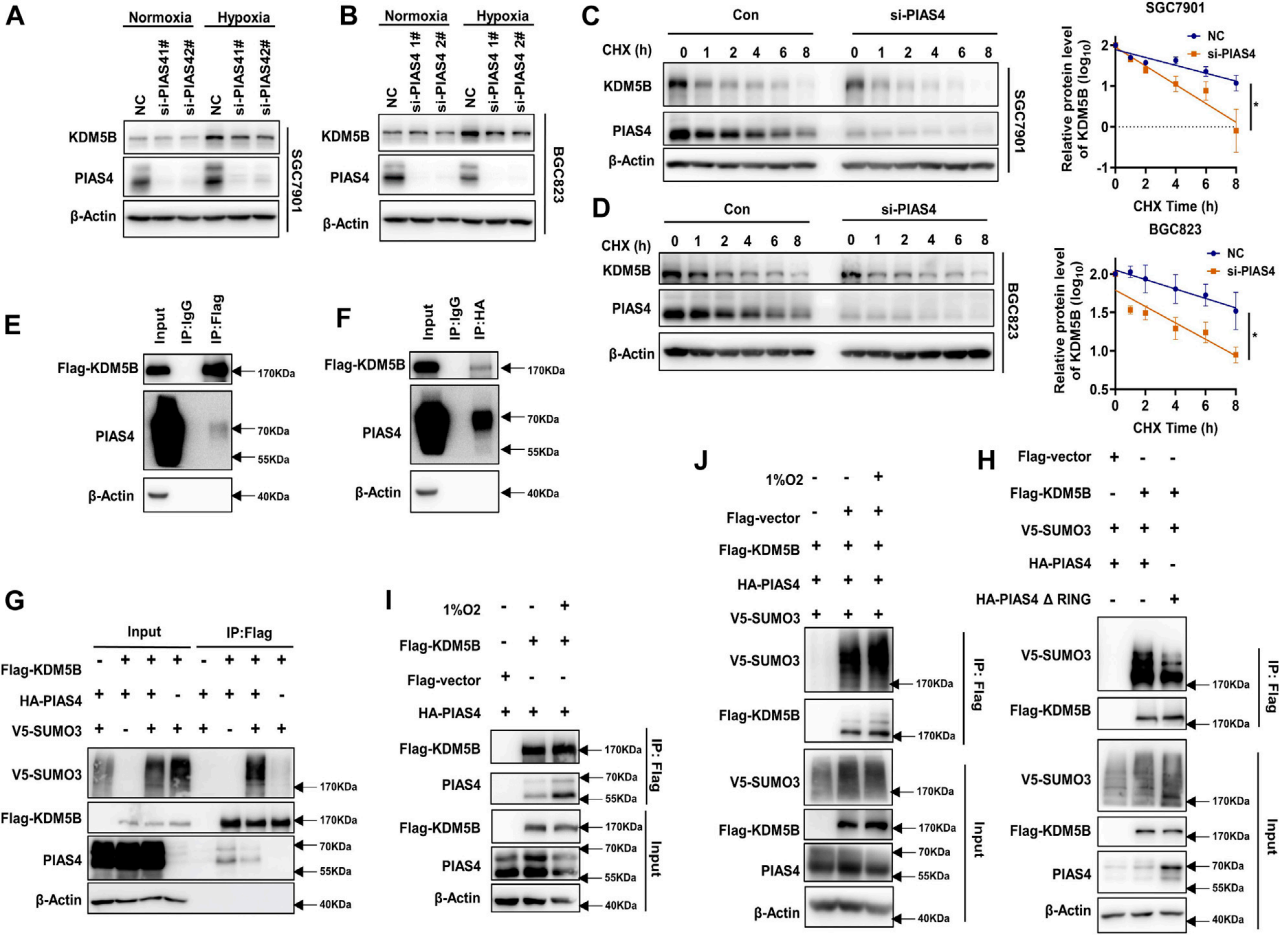
PIAS4 was the SUMO E3 ligase for hypoxia-induced KDM5B SUMOylation. **(A)** The protein level of the KDM5B protein in SGC7901 cells under normoxia or 6 h 1% O_2_ treatment with or without PIAS4 knockdown by siRNA for 48 h was analyzed by Western blotting. **(B)** SGC7901 was transiently transfected with PIAS4 siRNA for 48 h, later with cycloheximide (CHX) and 1% O_2_ treatment for the indicated times, and then the half-life of KDM5B was analyzed by Western blotting and followed by measurement by ImageJ. The *p*-value is derived from ANCOVA by comparing NC and si-PIAS4 (mean ± SD, n = 3, ANCOVA, *p < 0.05). **(C,D)** Co-IP was performed to detect the interaction between exogenous expression FLAG-KDM5B and HA-PIAS4 in HEK293T. The FLAG **(C)** and HA **(D)** antibody (IgG was used as negative control) was used for immunoprecipitation and the other for Western blotting analysis. **(E)** SUMOylation assay was employed to HEK293T and co-transfected with indicated plasmids for 48 h, followed by 1% O_2_ treatment for 6 h. The SUMOylation of KDM5B with or without the exogenous expression of the wild-type PIAS4 was determined by Western blotting. **(F)** The SUMOylation analysis of KDM5B in HEK293T co-transfected with the E3 activity inactivation mutant PIAS4 (HA-PIAS4 ΔRING (Sachdev et al., 2001) and the wild-type PIAS4 under hypoxia conditions. **(G)** The interaction between exogenous FLAG-KDM5B and HA-PIAS4 under hypoxia conditions was determined by co-IP in HEK293T. **(H)** The SUMOylation of KDM5B under normoxia and hypoxia (6 h 1% O_2_) was analyzed as indicated by the SUMOylation assay in HEK293T.

Next, we sought to determine which SUMO E3 ligase might be responsible for the SUMOylation of KDM5B protein. Since the PIAS family is the most well-known SUMO E3 ligase family (Rabellino et al., 2017), we performed an siRNA-based screen for potential SUMO E3 ligases regulating KDM5B protein stability (Supplementary Figure S4A). As shown, KDM5B protein level was reduced in GC cells after PIAS4 knockdown (Figures 3A,B). Similarly, the KDM5B protein level was much lower in *Pias4* ^*−/−*^ MEF cells than that of *Pias4* ^+/+^ MEF cells (Yu et al., 2018) (Supplementary Figure S4B). Meanwhile, the half-life of KDM5B protein was shortened in GC cells after PIAS4 knockdown under hypoxia conditions (Figures 3C,D). Furthermore, the interaction of PIAS4 with KDM5B could be confirmed by either co-IP assay (Figures 3E,F) or PLA assay (Supplementary Figure S4C), which also implicated their co-localization in the nucleus consistent with the distribution of KDM5B and PIAS4 in nuclear fraction (Supplementary Figure S4D). In addition, KDM5B SUMOylation could be enhanced by the wild-type PIAS4 (Figure 3G), but not its ligase-dead mutant (Figure 3H). More importantly, the interaction of KDM5B with PIAS4 and its SUMOylation was enhanced by hypoxia (Figures 3I,J). Collectively, PIAS4 was the E3 ligase responsible for hypoxia-induced SUMOylation of KDM5B.

### PIAS4-Mediated KDM5B SUMOylation Prevented It From Ubiquitination-Dependent Proteasomal Degradation

**FIGURE 4:**
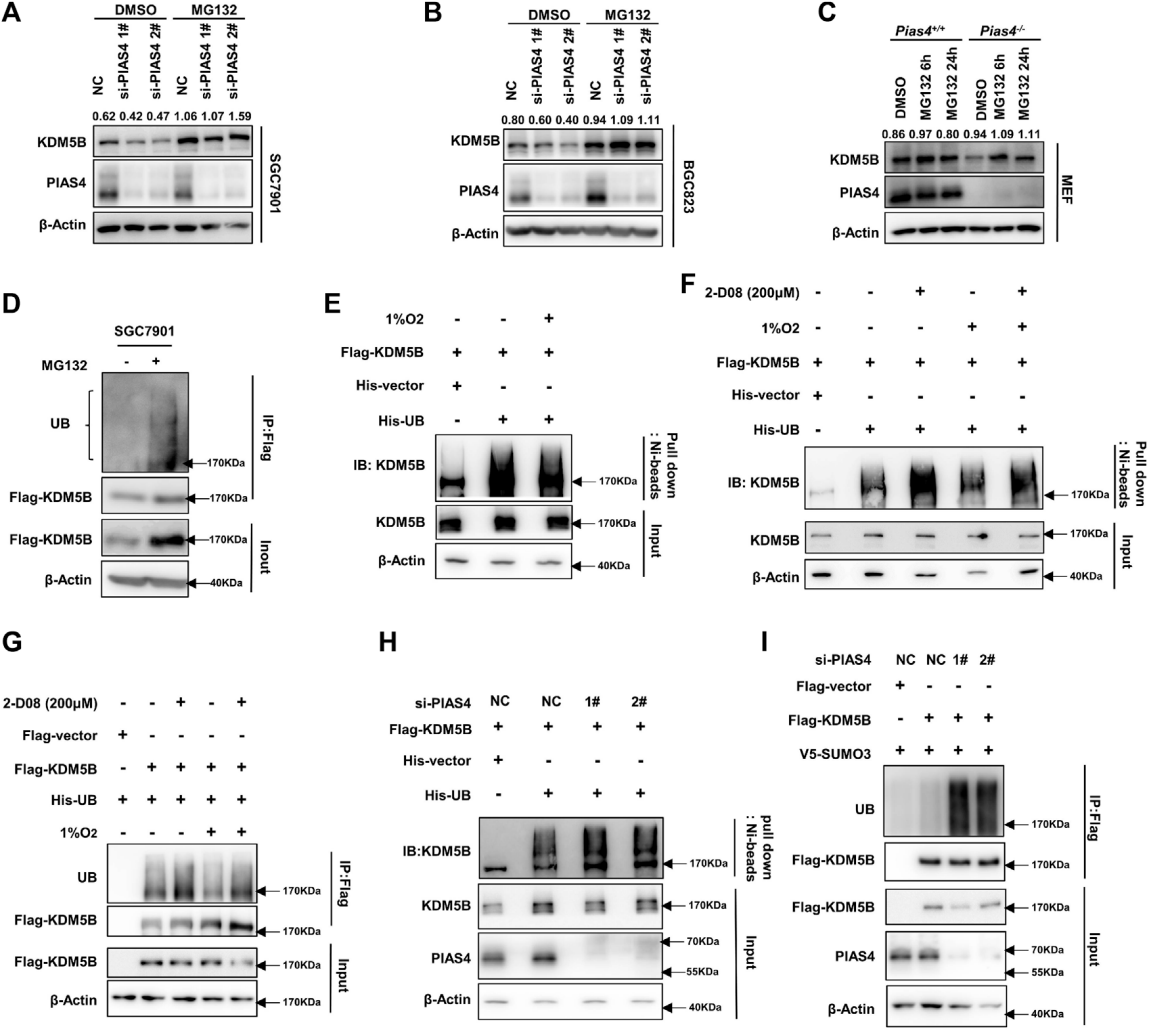
PIAS4-mediated KDM5B SUMOylation prevented it from ubiquitination-dependent proteasomal degradation. **(A and B)** KDM5B protein level in SGC7901 **(A)** and BGC823 **(B)** cells treated with MG132 (20 μM 6 h) after PIAS4 knockdown was detected by Western blotting. The quantification of KDM5B relative to β-actin was performed, and the values were labeled on the panel. **(C)** KDM5B protein level in *Pias4* ^*−/−*^ and *Pias4* ^+/+^ MEF cells after treatment of MG132 (20 μM 6h, 5 μM 24 h) under 1% O_2_ were determined by Western blotting. The quantification was performed as described before. **(D)** SGC7901 was transfected with FLAG-KDM5B for 48 h and the proteins immunoprecipitated by the FLAG antibody were analyzed with Western blotting. **(E)** Ni-beads pulldown assay was performed with HEK293T co-transfected FLAG-KDM5B, His-UB, or corresponding empty vector as indicated for 48 h later with 1% O_2_ and MG132 (20 μM) treatment for 6 h. KDM5B antibody was used to detect ubiquitination of KDM5B for Western blotting. **(F)** HEK293T was co-transfected with indicated plasmids for 48 h followed with 2-D08 (200 μM) for 24 h and 1% O_2_ and MG132 (20 μM) for 6 h before the Ni-beads pull-down assay was performed to determine the ubiquitination of KDM5B. **(G)** HEK293T cells co-transfected with indicated plasmids for 48 h followed with treatment as 4F, the immunoprecipitation with Triton buffer was performed to determine the ubiquitination of KDM5B. **(H)** HEK293T was co-transfected with Flag-KDM5B, His-UB, or corresponding empty vector as indicated and PIAS4 siRNA for 48 h. After the treatment of 1% O_2_ and MG132 (20 μM) for 6 h, the Ni-beads pull-down assay and immunoprecipitation with Triton buffer **(I)** were applied to analyze the ubiquitination of KDM5B.

As we previously reported, KDM5B protein turnover can be regulated by the ubiquitin–proteasome system (Xu et al., 2018). More interestingly, the sites of KDM5B predicted by online databases to be most likely modified by SUMOylation and ubiquitination were K242 and K278 (Supplementary Figures S5A,B). Furthermore, the same SUMOylation sites of KDM5B were identified in several SUMO proteomics studies (Hendriks et al., 2018), thus indicating a potential influence of SUMOylation on the ubiquitination-dependent degradation of KDM5B. Indeed, the proteasome inhibitor MG132 notably reversed the decrease in the KDM5B protein level caused by functional loss of PIAS4 in both GC cells and MEF cells (Figures 4A–C). While KDM5B underwent ubiquitination-dependent degradation in SGC7901 cells under normoxia conditions (Figure 4D), hypoxia significantly reduced the ubiquitination of KDM5B to prevent its subsequent degradation (Figure 4E), which was reversed by the SUMOylation inhibitor 2-D08 (Figures 4F,G). In accordance with that, the ubiquitination of KDM5B was increased after PIAS4 knockdown (Figures 4H,I). Taken together, these results suggested that PIAS4 SUMOylated KDM5B to prevent it from ubiquitination-dependent degradation under hypoxia.

### PIAS4 Upregulated KDM5B to Promote Hypoxia Adaption

**FIGURE 5:**
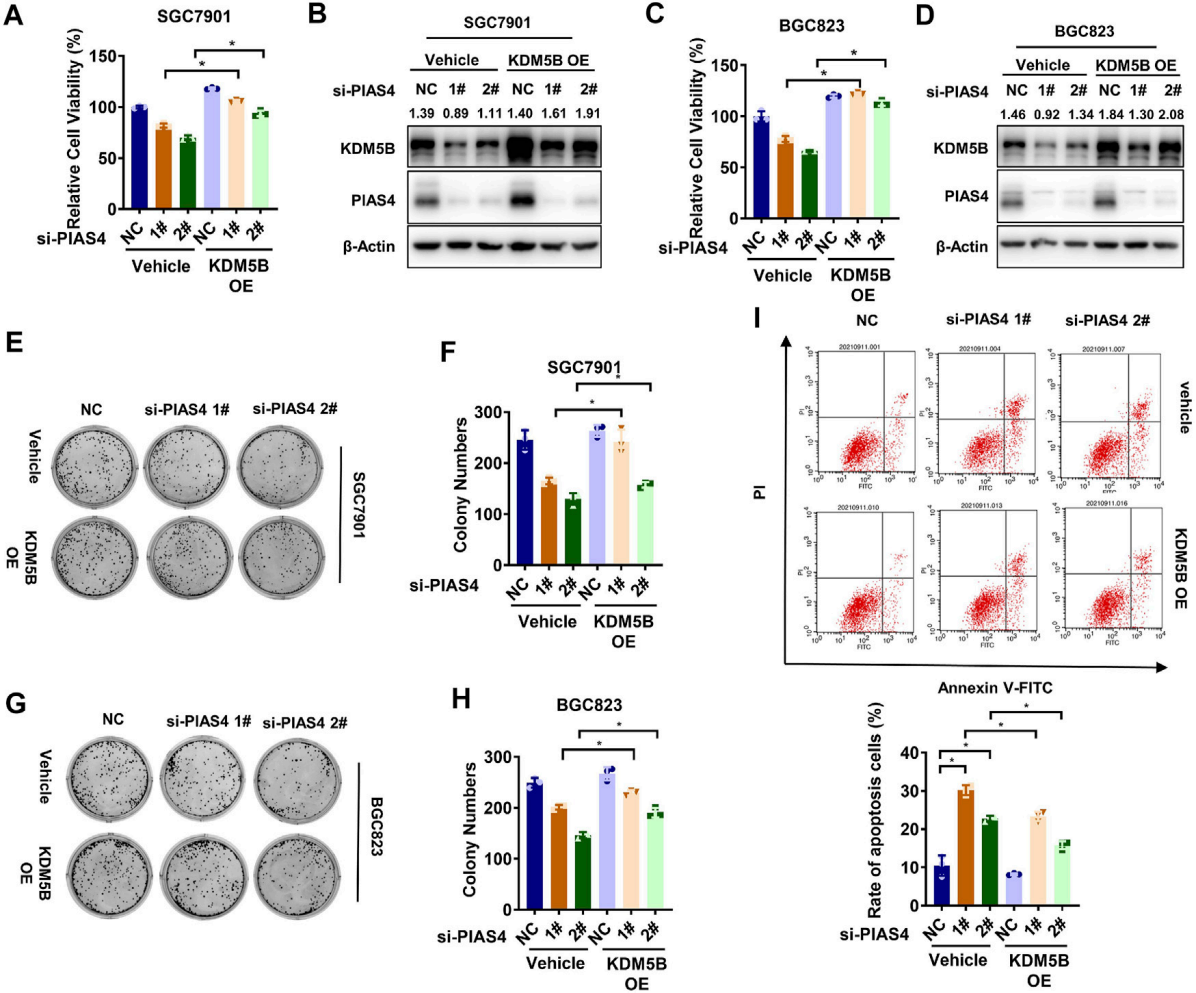
PIAS4 upregulated KDM5B to promote hypoxia adaption. **(A)** Relative cell viability of SGC7901 was determined by the CCK8 assay after co-transfected with PIAS4 siRNA or NC siRNA and FLAG-KDM5B or corresponding empty vector as indicated for 72 h under 1% O_2_ conditions. The NC group in vehicle was normalized to calculated the relative viability (mean ± SD, n = 3, one-way ANOVA, *p < 0.05). **(B)** The protein level of KDM5B was detected in SGC7901 cells after co-transfected with PIAS4 siRNA or NC siRNA and FLAG-KDM5B or corresponding empty vector as indicated under 1% O_2_ conditions by Western blotting. **(C)** Relative cell viability of BGC823 cells was determined by the CCK8 assay as described in **(A)**. **(D)** The protein level of KDM5B was detected in BGC823 cells as described in **(B)**. **(E)** SGC7901 cells was co-transfected with PIAS4 siRNA or NC siRNA and Flag-KDM5B or corresponding empty vector as indicated for 24 h before replating as single-cell suspension for colony formation for 10 days under 1% O_2_ conditions. **(F)** The number of colonies in **(E)** with more than 50 cells was counted and quantified (mean ± SD, n = 3, one-way ANOVA, *p < 0.05). **(G)** Colony formation assay was performed as described in **(E)** in BGC823 cells. **(H)** The number of colonies in **(G)** with more than 50 cells was counted and quantified (mean ± SD, n = 3, one-way ANOVA, *p < 0.05). **(I)** SGC7901 cells were co-transfected with PIAS4 siRNA or NC siRNA and FLAG-KDM5B or corresponding empty vector as indicated for 24 h, followed by 1% O_2_ treatment for 24 h. The cells were collected for PI/Annexin V flow cytometry analysis of cell apoptosis, and the percentage of apoptotic cells was statistically analyzed (mean ± SD, n = 3, one-way ANOVA, *p < 0.05).

It has been reported that PIAS4 plays critical roles in regulating the function or expression of proteins important to hypoxia response including VHL, the ubiquitination E3 ligase for the hypoxia-inducible factor-1α (HIF1α) (Cai et al., 2010; Chien et al., 2013). Thus, we speculated that PIAS4 might upregulate KDM5B to enable the adaption of GC cells to hypoxia. As expected, knocking down PIAS4 inhibited the viability (Figures 5A–D) and colony formation (Figures 5E–H) as well as induced apoptosis (Figure 5I) of GC cells under hypoxia, which could be partially restored by the overexpression of KDM5B.

### KDM5B Increases HIF1α Expression in Response to Hypoxia

**FIGURE 6:**
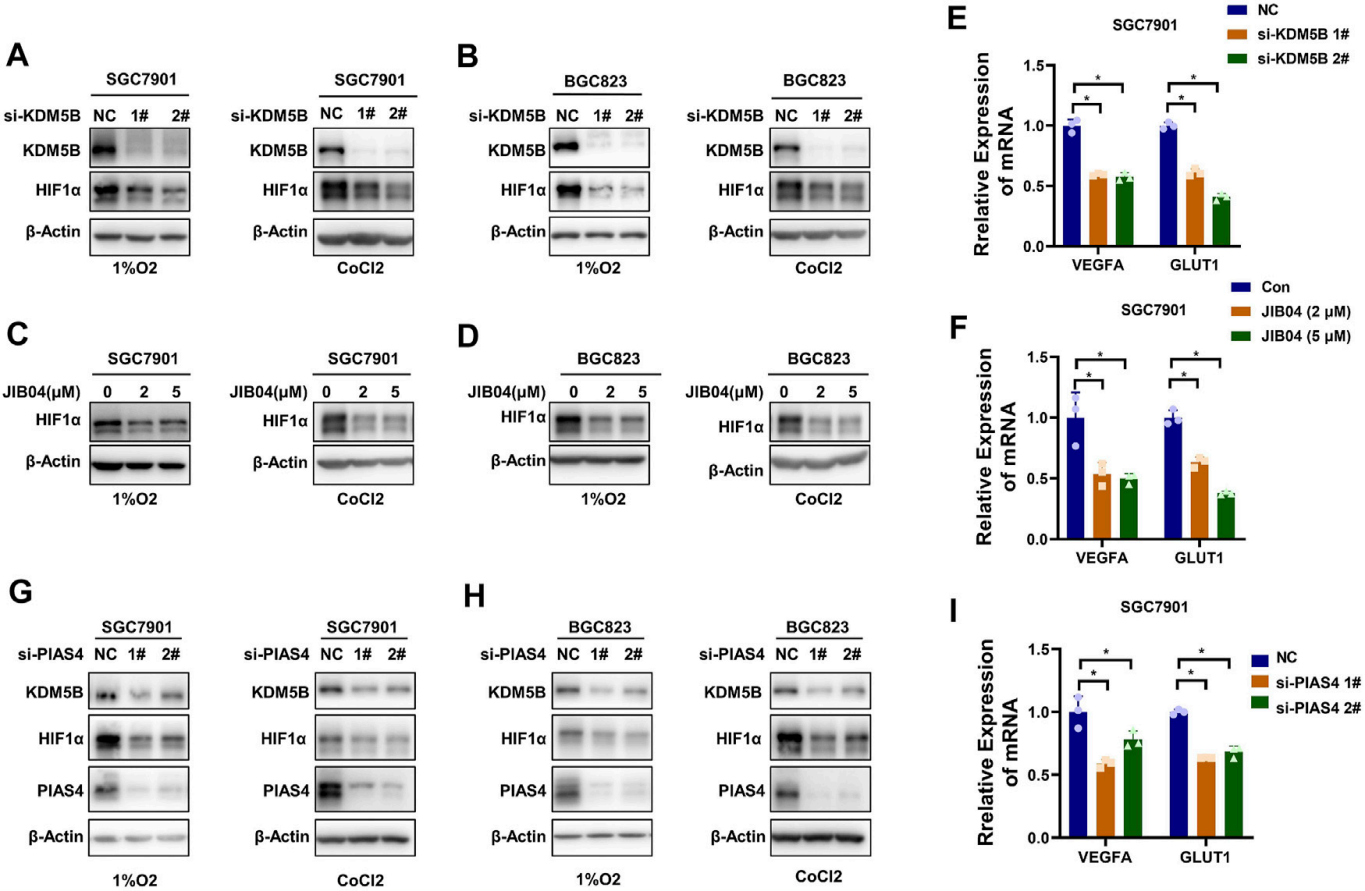
KDM5B increased HIF1α expression in response to hypoxia. **(A and B)** The protein levels of HIF1α in SGC7901 cells **(A)** and BGC cells **(B)** with treatment of 1% O_2_ **(left panel)** or CoCl_2_ (100 μM) **(right panel)** for 6 h after knocking down KDM5B with siRNA for 48 h were determined by Western blotting. **(C and D)** JIB04 was added to SGC7901 cells **(C)** and BGC cells **(D)** with two concentrations for 18 h before the treatment of 1%O_2_ **(left panel)** or CoCl_2_ (100 μM) for 6 h. The protein level of HIF1α was determined by Western blotting. **(E and F)** qPCR was conducted to determine the expression of HIF1α target genes (*VEGFA* and *GLUT1*) after knocking down KDM5B with siRNA **(E)** or JIB04 treatment **(F)** in SGC7901 cells cultured under 1%O_2_ for 12 h (mean ± SD, n = 3, one-way ANOVA, *p < 0.05). **(G and H)** The protein level of HIF1α in SGC7901 cells **(G)** and BGC823 cells **(H)** with treatment of 1%O_2_ **(left panel)** or CoCl_2_ (100 μM) for 6 h after knocking down PIAS4 with siRNA for 48 h was determined by Western blotting. **(I)** The HIF1α target gene (*VEGFA* and *GLUT1*) expression in SGC7901 cells after PIAS4 knockdown with siRNA was detected by qPCR (mean ± SD, n = 3, one-way ANOVA, *p < 0.05).

HIF1α is a critical factor in response to hypoxia by driving the transcription of multiple genes essential for hypoxia adaption (Semenza, 2000; Masoud and Li, 2015). Therefore, we explored the effect of KDM5B on HIF1α. As indicated, KDM5B knockdown impaired the HIF1α protein level in GC cells exposed to either 1% O_2_ or cobalt chloride (CoCl_2_) treatment, a well-known hypoxia-mimetic agent (Rana et al., 2019) (Figures 6A,B). And the consistent results were obtained by the treatment of KDM5B inhibitor JIB04 (Figures 6C,D). Meanwhile, the canonical downstream target genes of HIF1α (*VEGFA* and *GLUT1*) (Kim et al., 2016) were downregulated by the inhibition of KDM5B (Figures 6E,F). Similarly, PIAS4 knockdown induced the downregulation of HIFα protein (Figures 6G,H) and target gene expression as well (Figure 6I). These findings indicated that HIF1α was downstream of the KDM5B in response to hypoxia.

### Targeting KDM5B Overcomes Hypoxia Adaption *in vivo*

**FIGURE 7:**
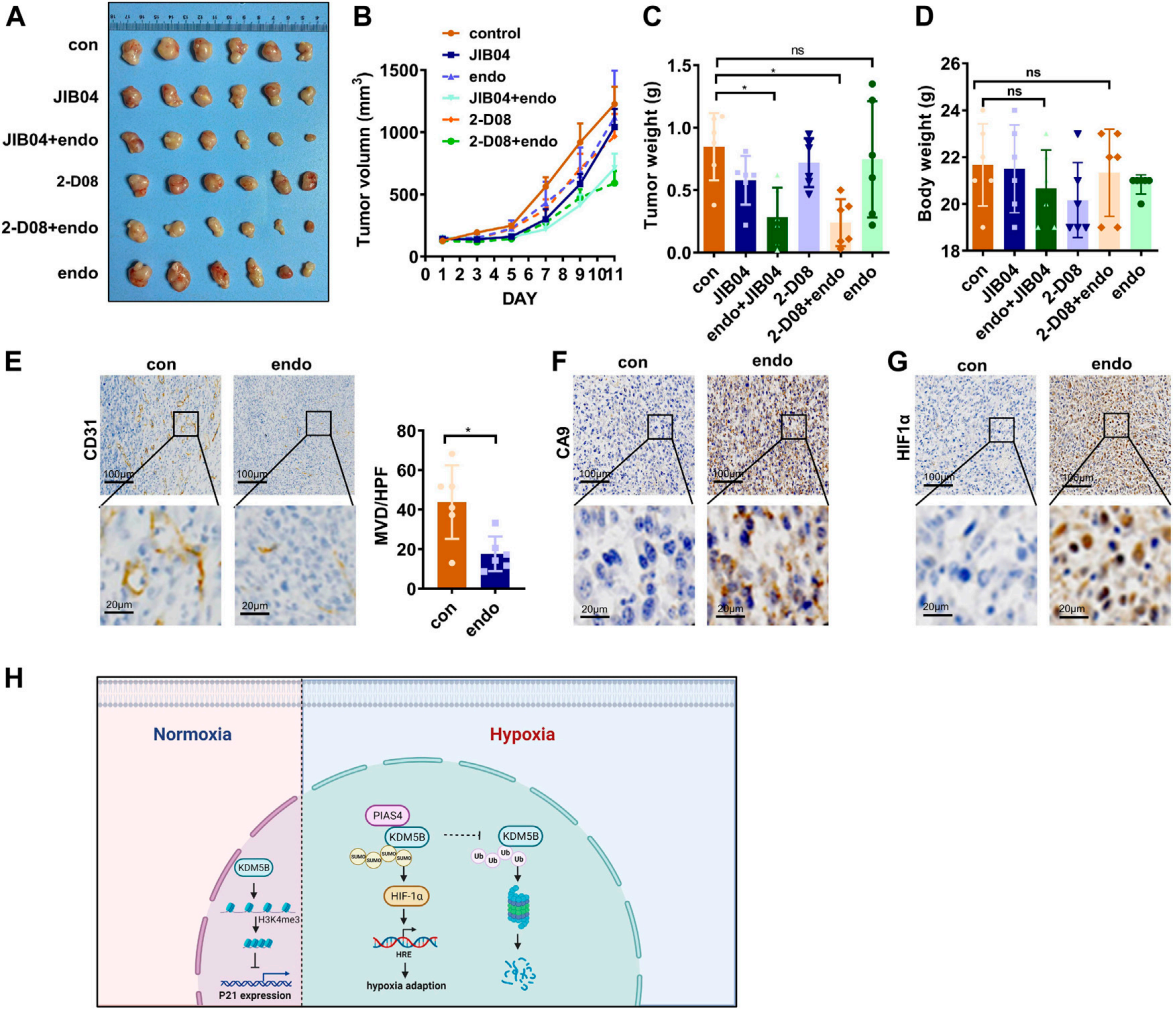
Targeting KDM5B to overcome hypoxia adaption *in vivo*. **(A–D)** MFC cells were subcutaneously injected into the flanks of nude mice to generate xenograft tumors (*n = 6*). And they were treated as indicated to evaluate the effect of JIB04, Endostar, and 2-D08 on tumorigenicity. The representative image **(A)**, tumor growth curve **(B)**, the tumor weight of xenograft at sacrifice **(C)**, and the body weight of mice during euthanasia **(D)** after drug injection for 11 days. **(E)** IHC staining with CD31 was applied to the section of the xenograft tumors from the control group and Endostar to evaluate the MVD. The presented images were shown **(left panel)**, and the brown areas represent vascular signals. And the MVD were compared in the two groups **(right panel)**. **(F and G)** IHC staining with CA9 **(F)** and HIF1α **(G)** was performed to reveal the hypoxia in the control and Endostar groups. **(H)** A schematic illustration of the model depicting the PIAS4-mediated SUMOylation protected KDM5B from ubiquitination-dependent proteasomal degradation of KDM5B to promote hypoxia adaption of gastric cancer.

Given the dependency of hypoxia adaption on KDM5B upregulation, targeting KDM5B might be an ideal strategy to overcome hypoxia adaption of cancer cells under anti-vascular treatment. Indeed, tumor growth in nude mice was only moderately attenuated after the treatment of Endostar, recombinant human endostatin used for the clinical treatment of patients with various cancers, including GC (Eder et al., 2002; Xu et al., 2013). Unfortunately, Endostar failed to inhibit the growth of GC *in vivo* (Figures 7A–D), although the antiangiogenesis effect and subsequent hypoxia was evident (Figures 7E–G). However, the addition of JIB04 or 2-D08 significantly improved the Endostar-induced inhibitory effect on tumor growth *in vivo* (Figures 7A–D). In line with the *in vitro* findings, these results highlighted that KDM5B inhibition successfully impaired GC cells to adapt hypoxia and improved antiangiogenesis therapy in GC.

## Discussion

In this study, we reported KDM5B was upregulated in gastric cancer; this upregulation of KDM5B enabled the survival of gastric cancer cells by inhibiting the transcription of CDKN1A (p21). It was further stabilized under hypoxia through PIAS4-mediated SUMOylation, which protected KDM5B from ubiquitination-dependent proteasomal degradation. Importantly, KDM5B promoted HIF1α-mediated hypoxia adaptation, and therefore represents a promising target to improve antiangiogenesis therapy.

It has been well-known that HIF1α is critical in hypoxia response. Recently, additional signaling pathways such as mTOR and UPR (unfolded protein response) pathways have been revealed to play important roles in response to hypoxia (Wouters and Koritzinsky, 2008). Moreover, mammalian cells contain a type of enzymes called 2-oxoglutarate–dependent dioxygenases (2-OGDDs) that take oxygen as the substrates for enzymatic reactions. Many 2-OGDDs act on remodeling chromatin structure as methylation erasers such as DNA demethylases TETs and histone lysine demethylases, thus linking oxygen concentration with epigenetic reprogramming (Batie et al., 2019; Chakraborty et al., 2019). Likewise, hypoxia caused DNA hypermethylation in cancer cells by reducing TET activity (Thienpont et al., 2016). Furthermore, lysine methylation of HIF-1α protein regulated by histone demethylase LSD1 influenced its stability independent of its proline hydroxylation (Kim et al., 2016; Lee et al., 2017). Although KDM5B was well-known to predominantly affect gene expression through demethylating histones, KDM5B and other family members may demethylate non-histone proteins under some specific conditions. Whether such functions might be relevant to hypoxia adaption remains to be further explored.

It would be valuable to understand the cellular response to hypoxia more complete and design effective strategies for hypoxia-targeting therapies such as antiangiogenesis approaches, by clarifying the relevance and regulation of more signaling pathways associated with hypoxia response. In this study, we found that SUMOylation-dependent stabilization of KDM5B is important to confer hypoxia adaption in gastric cancer.

Lysine SUMOylation is a reversible post-translational modification, which efficiently regulates the localization and function of various proteins (Wilson, 2017). For instance, HIF-1α was SUMOylated by Cbx4 to transactivate the transcription of VEGF (Li et al., 2014), which could be reversed by RSUME and SENP1 dependent-deSUMOylation (Carbia-Nagashima et al., 2007; Cheng et al., 2007). Interestingly, PIAS4-mediated SUMOylation of VHL blocked its ubiquitination-dependent degradation, thus upregulating the HIF-1α protein level (Cai et al., 2010; Chien et al., 2013). Here, we found KDM5B as another downstream target of PIAS4 in the hypoxia response. The growth inhibition induced by PIAS4 inhibition could be partially rescued by the overexpression of KDM5B (Figure 5), highlighting the importance of KDM5B in the hypoxia response.

SUMO1-4 are four different SUMO family members present in mammals, and SUMO1-3 are conjugated and play critical role in biological functions of proteins (Eifler and Vertegaal, 2015). Among them, SUMO2 and SUMO3 have a high degree of homology in sequence and have major similar functional properties. However, they were distinguished in many research studies due to their different actions. For example, the conjugation of SUMO2, but not SUMO3, to PCNA was induced to facilitate chromatin remodeling (Li et al., 2018). And the deficiency of SUMO3, but not SUMO2, conferred functional deficiency of homology-directed repair (HDR) of DNA double-strand break (Bodo et al., 2019). SUMO3 modification was the only form of MAVS induced by poly (dA:dT) treatment in human keratinocytes (Choi et al., 2020). Moreover, in arabidopsis, the levels of SUMO1 and SUMO2, but not SUMO3, conjugates increased substantially under stress conditions (Kurepa et al., 2003). And in this study, we found that SUMO3 was dominant in the modification of KDM5B, and increased under hypoxia. However, the mechanism underlying the selective modification of SUMO2 or SUMO3 was unknown, and additional studies are needed to explore the distinct functions and regulations of SUMO2 and SUMO3.

Nevertheless, the crosstalk between SUMOylation and ubiquitination was extensively explored recently. The same proteins can be conjugated to SUMO and ubiquitin for antagonistic, synergistic or other outcomes, demonstrating the complexity of the cellular signaling networks. For example, the dual modification at Lys^21^ of IκBα led to opposite results to its stabilization and subsequent NF-κB activation (Desterro et al., 1998). In contrast, these two modifications formed a cooperative relationship in a sequential manner under some situations. In response to DNA damage, hybrid SUMO–ubiquitin chains can be synthesized by RNF4, a SUMO-targeted ubiquitin E3 ligase, thus recruiting RAP80 and BRCA1 to the sites of DNA damage (Guzzo et al., 2012). In our study, the SUMOylation and ubiquitination of KDM5B seem to be exclusive for the stabilization of KDM5B protein. However, the underlying mechanisms including verification of the SUMOylation- and ubiquitination-modified sites and functions as well as identification of the ubiquitin E3 ligase warrant further investigations.

Despite the important role of angiogenesis in GC, clinical trials of anti-vascular therapy in GC have not yielded encouraging results so far (Raimondi et al., 2018). Therefore, targeting hypoxia adaption could be necessary to improve the clinical efficacy of anti-vascular therapy in GC. Our study indicated that KDM5B was a key regulatory factor in hypoxia adaption. Targeted inhibition of KDM5B might be considered in combination of anti-vascular therapy in GC. As KDM5B was upregulated to play an oncogenic role in multiple cancers, many chemical inhibitors of KDM5B have been extensively evaluated for their potential in targeted therapy of human cancer (Zheng et al., 2019; Fu et al., 2020). The combination of these inhibitors with anti-vascular therapy could be taken into consideration while designing upcoming clinical trials.

## Conclusion

In conclusion, PIAS4-mediated SUMOylation stabilized KDM5B protein by disturbing ubiquitination-dependent proteasomal degradation to overcome hypoxia adaption. Targeting SUMOylation-dependent KDM5B upregulation might be considered when antiangiogenic therapy was applied in cancer treatment.

## Data Availability

The original contributions presented in the study are included in the article/Supplementary Material, further inquiries can be directed to the corresponding authors.

## Abbreviations

KDM5B, lysine-specific demethylase 5B; GC, gastric cancer; CDKN1, cyclin-dependent kinase inhibitor 1; SUMO, small ubiquitin-related modifier; PIAS4, protein inhibitor of activated STAT 4; qPCR: quantitative real-time PCR; IHC: immunohistochemistry; WB, Western blotting; ChIP, chromatin immunoprecipitation; co-IP, co-immunoprecipitation; PLA, *in situ* proximity ligation assay; TMA, tissue microarray; KEGG, Kyoto Encyclopedia of Genes and Genomes; DEG, differential expressed gene; TCGA, The Cancer Genome Atlas; PTM, post-translation modification; MVD, microvascular density; HDR, homology-directed repair; HIF1α, hypoxia-inducible factor-1α.
