# Supplementary material for: Hypoxia Stimulates SUMOylation-Dependent Stabilization of KDM5B
Source: Front Cell Dev Biol. 2021 Dec 17;9:741736. doi: 10.3389/fcell.2021.741736 (PMC8719622; doi:10.3389/fcell.2021.741736)
Supplement: Supplementary file 1 [file DataSheet1.docx]

***Supplementary Material***

**Supplementary Figures & Figure legends**


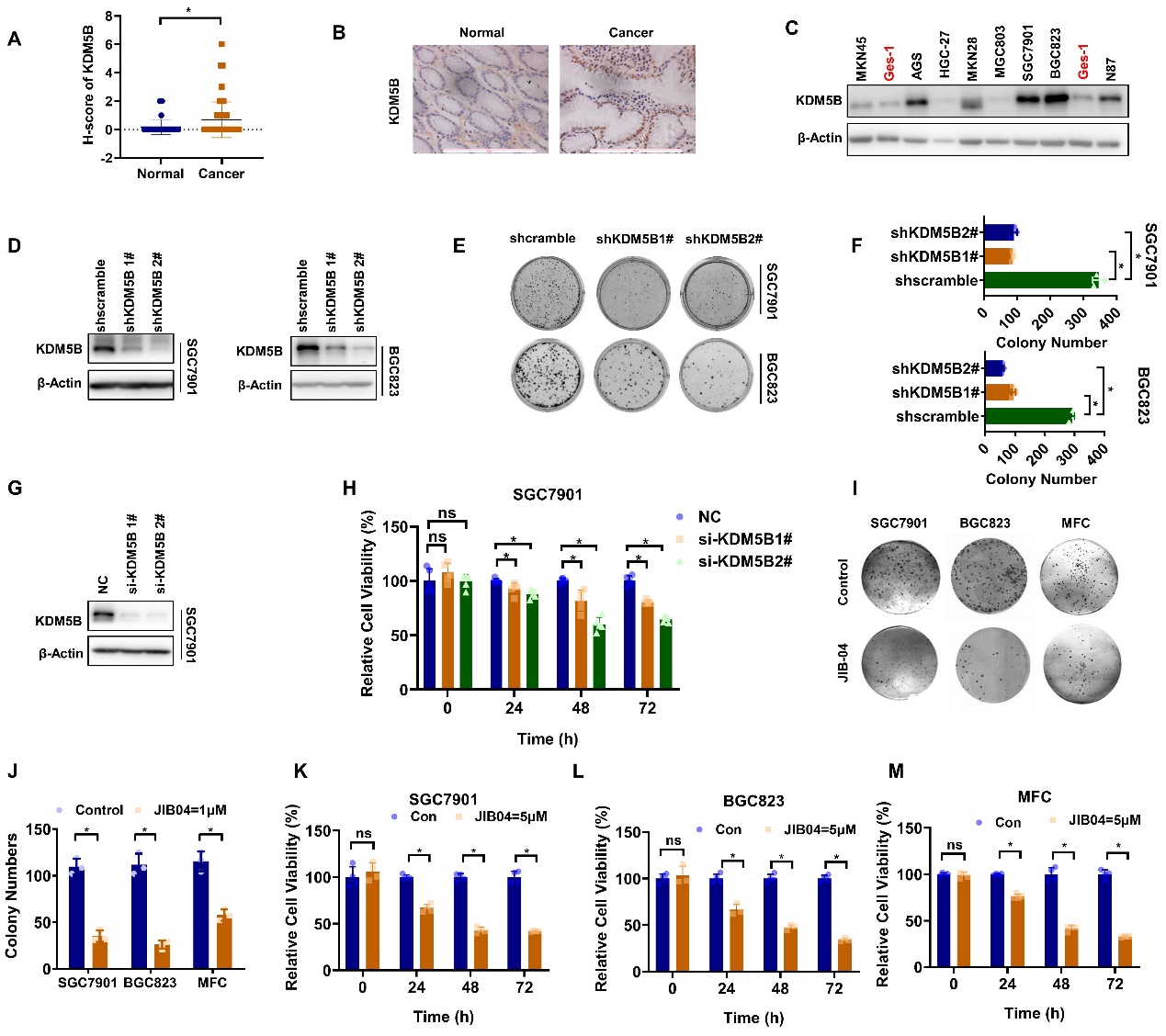


**Supplementary Figure 1.**

**KDM5B was upregulated to promote cell proliferation in GC**

**(A)** Immunohistochemical staining of KDM5B in tissue microarray containing 77 GC tissue and corresponding adjacent normal tissues. The H-score was calculated and analyzed. (*n=71,* *Wilcoxon signed-rank test, *p<0.05*). **(B)** Represented photos of KDM5B IHC in GC tissue and normal tissue. **(C)** Expression of KDM5B protein in GC cell lines and GES-1 cell line was analyzed by Western blotting. **(D)** The knocking down of KDM5B by shRNA in SGC7901 and BGC823 cells was verified by Western blotting. **(E)** Plate colony formation assay for SGC7901 and BGC823 cells transfected with shKDM5B or scramble control (shscramble) containing lentiviral particles. **(F)** The number of colonies with more than 50 cells was counted and quantified (*mean ± SD, n = 3, one-way ANOVA, *p<0.05*). **(G)**The knocking down of KDM5B by siRNA in SGC7901 cells was verified by Western blotting. **(H)** Relative cell viability of SGC7901 cells at 24h, 48h and 72h after transient transfection with KDM5B or negative control (NC) siRNA was measured with CCK8 assay (*mean ± SD, n = 3, one-way ANOVA, *p<0.05*). **(I, J)** SGC7901, BGC823 and MFC cells were treated with JIB04 (1μM) and colony formation was analyzed (*mean ± SD, n = 3, one-way ANOVA, *p<0.05*). **(K-M)** Relative cell viability of SGC7901, BGC823, MFC cells at 24h, 48h and 72h after treatment of JIB04 (5μM) was determined with CCK8 assay (*mean ± SD, n = 3, one-way ANOVA, *p<0.05*).

**
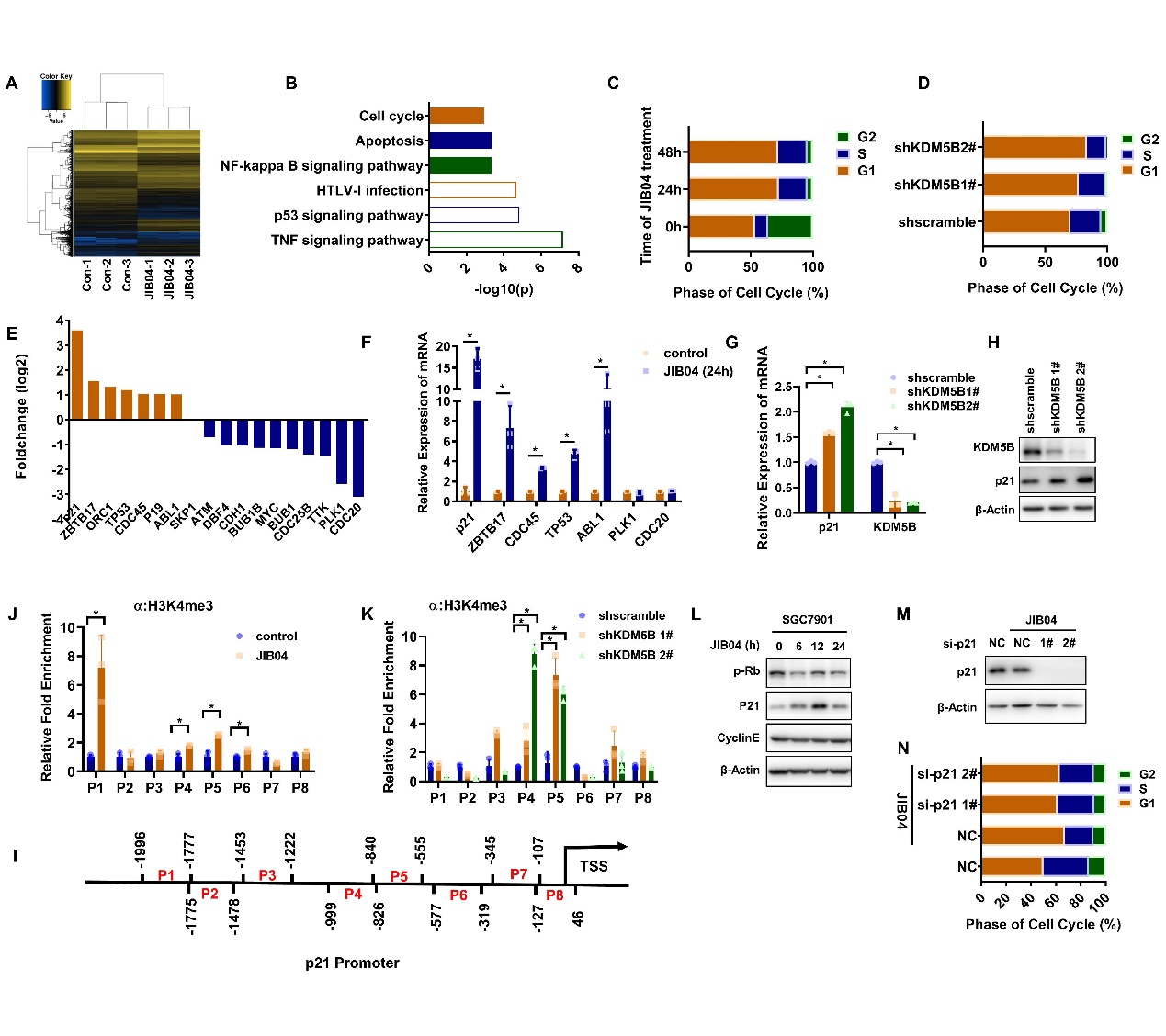
**

**Supplementary Figure 2**

**KDM5B repressed p21 transcription to induce cell cycle arrest at G1**

Cluster analysis **(A)** and KEGG analysis **(B)** of the mRNA profile on JIB-04-treated GC cells. **(C)** Flow cytometry analysis of cell cycle inSGC7901 cells before and after treatment of JIB04 (5μM) at 24 and 48 hours. **(D)** Flow cytometry analysis of cell cycle in BGC823 shKDM5B knockout cells. **(E)** Significantly differentially expressed genes involved in cell cycle regulation. **(F)** qPCR was performed to verify the result of sequencing. **(G, H)** p21 expression was detected by qPCR **(G)** and Western blotting in BGC823 shKDM5B knockout cells **(H)**. **(I)** a schematic diagram of the p21 promoter where primers for CHIP assay were designed. **(J)** The binding of tri-methylation H3K4 to p21 promoter in SGC7901 cells before and after JIB04 treatment was assessed by ChIP assay. **(K)** The occupancy of tri-methylation H3K4 on p21 promoter in BGC823 shKDM5B knockout cells was determined by CHIP assay**. (L)** The expression of G1 phase regulatory protein in SGC7901 cells treated with JIB04 (5μM) for various times were evaluated by Western blotting. **(M)** Western blotting was performed to prove the knockdown of p21. **(H)** Flow cytometry analysis of cell cycle in SGC7901 cells with the treatment as indicated.

**
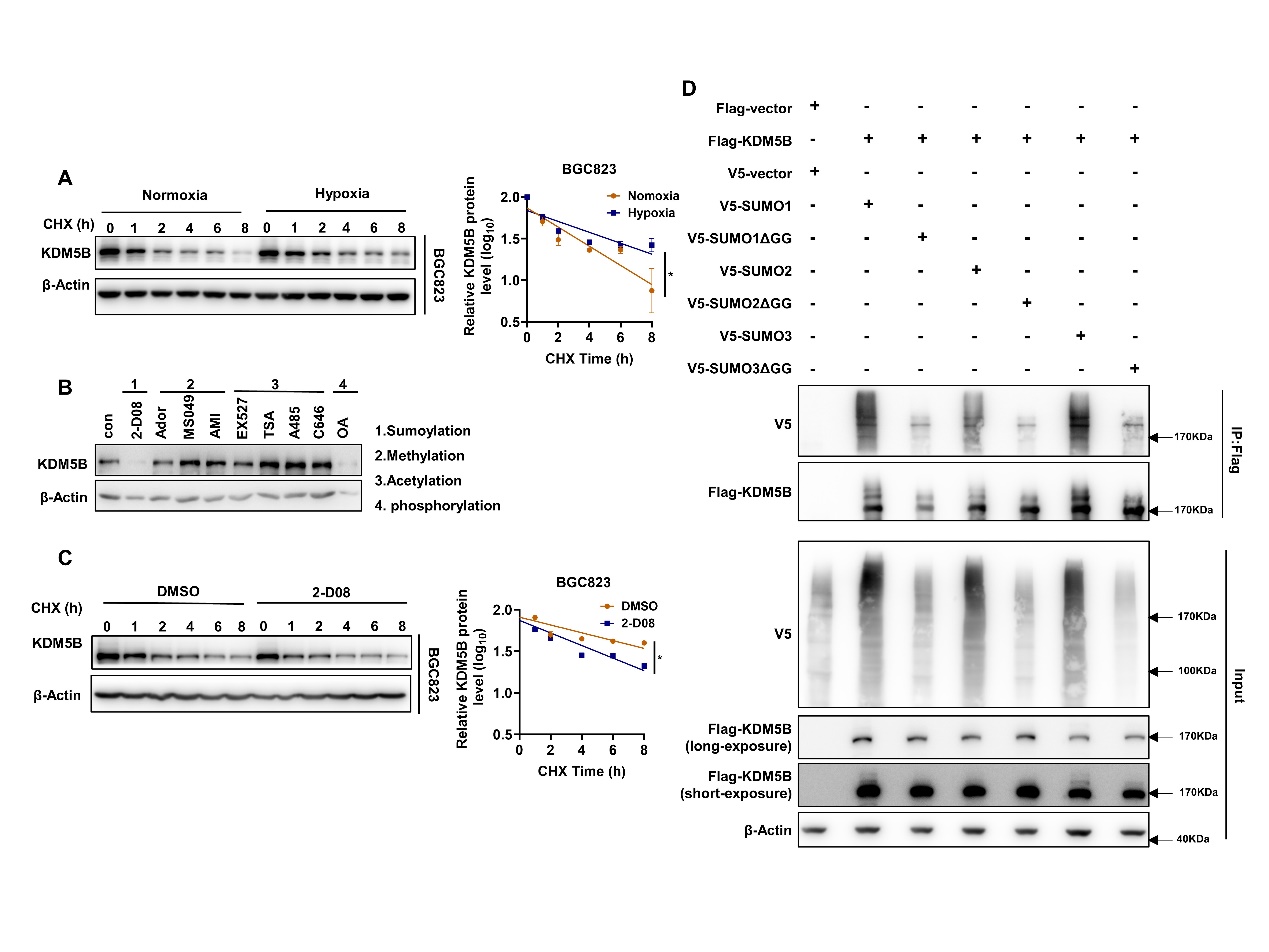
**

**Supplementary Figure 3**

**Hypoxia induced SUMO3-dependent SUMOylation and subsequent stabilization of KDM5B**

**(A)** The half-life of KDM5B protein in BGC823 cells under normoxia and hypoxia (1%O_2_) was determined by CHX assay. The relative KDM5B protein expression was quantified by ImageJ (*mean ±* *SD, n = 3,* ANCOVA analysis*, *p<0.05*). **(B)** KDM5B expression in SGC7901 cells treated with various inhibitors as indicated，2-D08 (200μM), ADOX (20μM), MS049 (10μM), AMI-1 (10μM), EX527 (100nM), TSA (1μM), A485 (10nM), C646 (10μM), and okadaicacid (OA) (10nM) for 24h were determined by Western blotting. **(C)** The half-life of KDM5B protein in BGC823 cells under hypoxia (1%O_2_) with 2-D08 (200μM) or DMSO treatment was determined by CHX assay. The relative KDM5B protein expression was quantified by ImageJ (*mean ± SD, n = 3,* ANCOVA analysis*, *p<0.05*). **(D)** HEK293T co-transfected with indicated plasmids for 48h under normoxia, and the SUMOylation assay with Flag antibody was performed followed by western blot analysis.

**
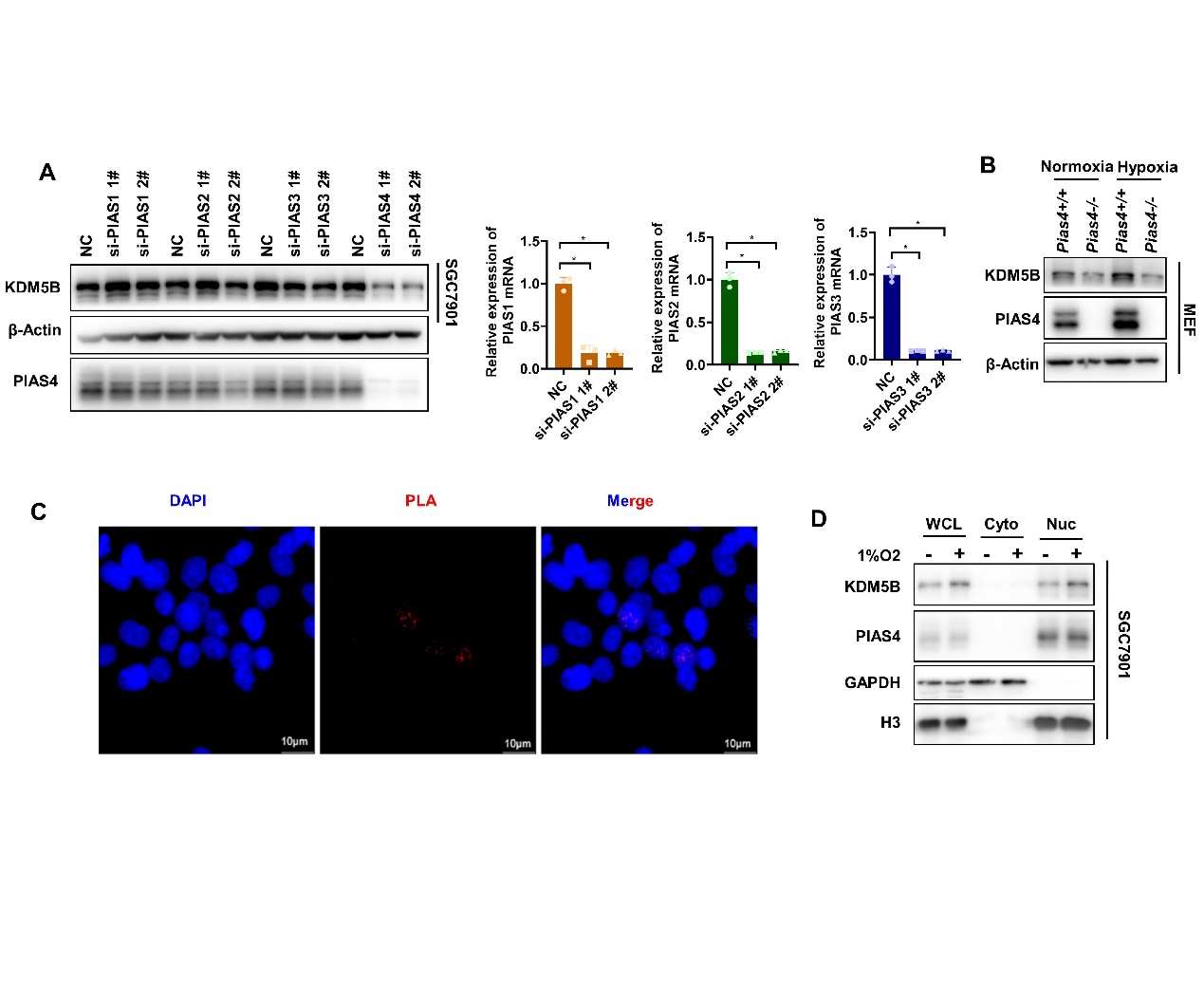
**

**Supplementary Figure 4**

**PIAS4 was the SUMO E3 ligase for hypoxia-induced KDM5B SUMOylation.**

**(A)** The protein level of KDM5B was determined by Western blotting with or without the PIAS1/2/3/4 knocking down by siRNA with 1%O_2_ treatment for 6h. **(B)** Western blotting analysis of the protein expression of KDM5B in *Pias4*+/+ and *Pias4-/-* MEF under normoxia and 1%O_2._for 6h. **(C)** PLA assay was performed withing Flag and PIAS4 antibody after 293T co-transfected with Flag-KDM5B and HA-PIAS4 for 48h. The red PLA spots in the nucleus indicated a positive PLA signal, suggesting interactions between the two proteins. **(D)** The nuclear and cytosol fractions were isolated using the Nuclear-Cytosol Extraction Kit (Thermo, USA), then the distribution of KDM5B and PIAS4 was analyzed by western blotting (H_3_ and GAPDH were used as marker of nuclear and cytosol fraction separately).


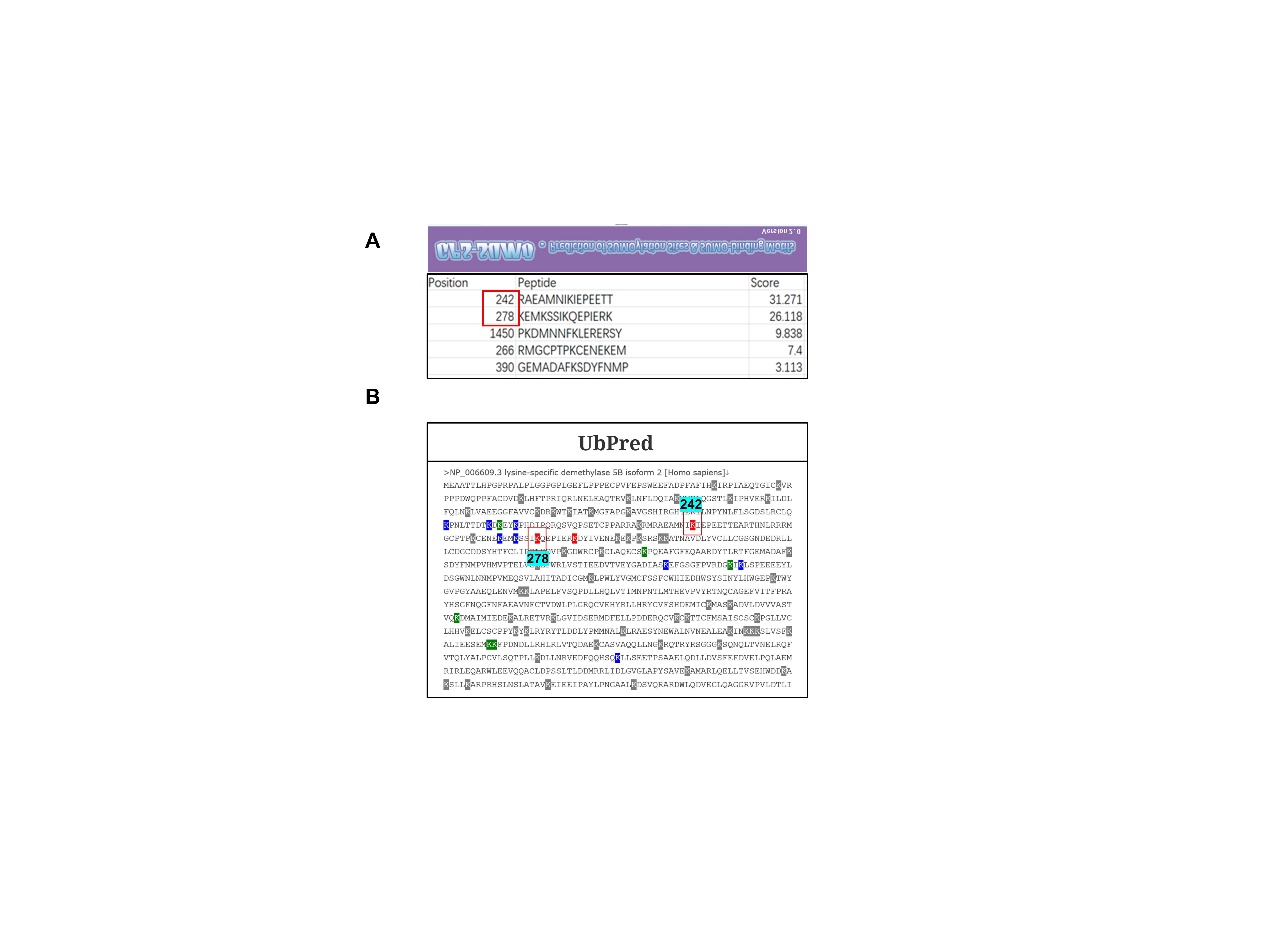


**Supplementary Figure 5**

**PIAS4-mediated KDM5B SUMOylation prevents it from ubiquitination-dependent proteasomal degradation**

**(A)** The SUMOylation modification sites of KDM5B were predicted by GPS-SUMO based on the SUMO modification consensus motif. K242 and K278 were the top 2 sites which were the most potential sites modified by SUMOylation. **(B)** The ubiquitination sites of KDM5B were predicted by UbPred. K242 and K278 were in the red highlight, means the strongest possibility be ubiquitinated at these 2 sites.

**Supplementary Tables**

**Supplementary Table 1**

| **Name** | **Sequence** | **supplier** |
| --- | --- | --- |
| siKDM5B-1# | GAGGGCAUUAUGAACGAAUTT  AUUCGUUCAUAAUGCCCUCTT | Gene Pharma Company (Shanghai, China) |
| siKDM5B-2# | GGAGACUAGUAAGCACUAUTT  AUAGUGCUUACUAGUCUCCTT | Gene Pharma Company (Shanghai, China) |
| siPIAS1-1# | GGAACUAAAGCAAAUGGUUTT  AACCAUUUGCUUUAGUUCCTT | Gene Pharma Company (Shanghai, China) |
| siPIAS1-2# | GCAGCCUGGUUUCUUCCAATT  UUGGAAGAAACCAGGCUGCTT | Gene Pharma Company (Shanghai, China) |
| siPIAS2-1# | CCCUGCGGUUCAGAUUAAATT  UUUAAUCUGAACCGCAGGGTT | Gene Pharma Company (Shanghai, China) |
| siPIAS2-2# | GCCUAUGAAAGUCUAAUAUTT  AUAUUAGACUUUCAUAGGCTT | Gene Pharma Company (Shanghai, China) |
| siPIAS3-1# | GCAAGUGCAGCAGAUUCUUTT  AAGAAUCUGCUGCACUUGCTT | Gene Pharma Company (Shanghai, China) |
| siPIAS3-2# | CCCUUUAUCUACAGAUGAATT  UUCAUCUGUAGAUAAAGGGTT | Gene Pharma Company (Shanghai, China) |
| siPIAS4-1# | GCUGAAGCCCACCGAAUUATT  UAAUUCGGUGGGCUUCAGCTT | Gene Pharma Company (Shanghai, China) |
| SiPIAS4-2# | GCUCUACGGAAAGUACUUATT  UAAGUACUUUCCGUAGAGCTT | Gene Pharma Company (Shanghai, China) |

**siRNA sequences used for knockingdown**

**Supplementary Table 2**

**Primer sequences used for clone**

| PIAS4 **Δ**RING-C337S | F:5'-CTCTGCACGGGAGGGCACCGAGAG-3'  R:5'-CTCTCGGTGCCCTCCCGTGCAGAG-3' |
| --- | --- |
| PIAS4 **Δ**RING-C342S | F:5'-GCAGGTGTGCGGAGGTCTCTGCACGG-3'  R:5'-CCGTGCAGAGACCTCCGCACACCTGC-3' |
| PIAS4 **Δ**RING-H344A | F:5’-TGCAGAGACCTCCGCAGCCCTGCAGTGCTTTG-3’  R:5'-CAAAGCACTGCAGGGCTGCGGAGGTCTCTGCA-3' |
| PIAS4 **Δ**RING-C347S | F:5'-ACACAGCATCAAAGGACTGCAGGGCTGCG-3'  R5’-CGCAGCCCTGCAGTCCTTTGATGCTGTGT-3’ |

**Supplementary Table 3**

**Primer sequences used for qPCR**

| HUMAN-P21 | F:5’-CTGGAGACTCTCAGGGTCGAAA-3’  R:5’-GATTAGGGCTTCCTCTTGGAGAA-3’ |
| --- | --- |
| HUMAN-ZBTB17 | F:5’-TGTAACCCCTCCCTCCAAGC-3’  R:5’-GTCTAGCACACAGCTCTGAACG-3’ |
| HUMAN-CDC45 | F:5’-CTTGAAGTTCCCGCCTATGAAG-3’  R:5’-GCATGGTTTGCTCCACTATCTC-3’ |
| HUMAN-TP53 | F:5’-CCTGGTCCTCTGACTGCTCT-3’  R:5’-GTGTAGGAGCTGCTGGTGCA-3’ |
| HUAMN-ABL1 | F:5’-AGGAGCTCTCATGGGTGAACA-3’  R:5’-GTTCTCCCCTACCAGGCAGTT-3’ |
| HUMAN-PLK1 | F:5’-AAAGAGATCCCGGAGGTCCTA-3’  R:5’-GGCTGCGGTGAATGGATATTTC-3’ |
| HUMAN-CDC20 | F:5’-GCACAGTTCGCGTTCGAGA-3’  R:5’-CTGGATTTGCCAGGAGTTCGG-3’ |
| HUMAN-KDM5B | F:5’-AATCAAACTGAGCCACCCCA-3’  R:5’-CAGTCCACCTCATCTCCTTCTG-3’ |
| HUMAN-VEGFA | F:5’-TACTGCCGTCCGATTGAGA-3’  R:5’-GCTGGCTTTGGTGAGGTTT-3’ |
| HUMAN-GLUT1 | F:5’-ATTGGCTCCGGTATCGTCAAC-3’  R:5’-GCTCAGATAGGACATCCAGGGTA-3’ |
| HUMAN-ACTINB | F:5’-ACTCTTCCAGCCTTCCTTCC-3’  R:5’-CGTCATACTCCTGCTTGCTG-3’ |
| HUMAN-p21-chip-p1 | F:5’-AGTGTGGCCAAAGGATCTGA-3’  R:5’-ACTGAGATTTGCAGCAGACAC-3’ |
| HUMAN-p21-chip-p2 | F:5’-GCCCATTAATATTATAGGTCTGGC-3’  R:5’-CAATGCTGGC CTCGAAG-3’ |
| HUMAN-p21-chip-p3 | F:5’-GCCTCTCTTCAAACATTGTACAAG-3’  R:5’-TCTATGAGAGTCCTTGTGGGC-3’ |
| HUMAN-p21-chip-p4 | F:5’-GGAGTCTCACTCTGTCACCCA-3’  R:5’-AATATGGTG AAACCCCGTC TC-3’ |
| HUMAN-p21-chip-p5 | F:5’-AGACGGGGTTTCACCATATTG-3’  R:5’-GATTACAGGCATGCACCACC-3’ |
| HUMAN-p21-chip-p6 | F:5’-ATGGTGGTGCATGCCTGTA-3’  R:5’-TCAGCTTTCAGAGGAATTCACC-3’ |
| HUMAN-p21-chip-p7 | F:5’-TGAAGGTGAATTCCTCTGAAAGC-3’  R:5’-GACAAGAGTGCCCAGTCCAG-3’ |
| HUMAN-p21-chip-p8 | F:5’-CTGGACTGGGCACTCTTGTC-3’  R:5’-GACAAAATAGCCACCAGCCTC-3’ |
